# RETRACTION: Chemical Characterization and Comparative Biological Activities of *Spinacia oleracea* and *Basella rubra* Leaf Extracts

**DOI:** 10.1155/bri/9859376

**Published:** 2026-06-18

**Authors:** Biochemistry Research International

RETRACTION: F. Shahid, M. A. Razzaq, A. Sajid, et al., “Chemical Characterization and Comparative Biological Activities of *Spinacia oleracea* and *Basella rubra* Leaf Extracts,” *Biochemistry Research International* 2025, no. 1 (2025), 1–10, https://doi.org/10.1155/bri/7140041.

The above article, published online on 19 December 2025 in Wiley Online Library (https://wileyonlinelibrary.com), has been retracted by agreement between the journal Editor‐in‐Chief, Yoel Klug; and John Wiley & Sons Ltd.

The article has been retracted due to significant concerns regarding the reliability of the authorship relating to the stated contributions of individual authors. We have seen evidence leading to concerns that the article may not have been produced as described and it is therefore retracted.

All authors responded with agreement when they were notified of its retraction, except Z. Hidayatallah who remained unresponsive.

